# The Human Neck is Part of the Musculoskeletal Core: Cervical Muscles Help Stabilize the Pelvis During Running and Jumping

**DOI:** 10.1093/iob/obac021

**Published:** 2022-06-02

**Authors:** Alicia M Boynton, David R Carrier

**Affiliations:** Division of Biological Science, University of Utah, Salt Lake City, Utah 84112, USA; Division of Biological Science, University of Utah, Salt Lake City, Utah 84112, USA

## Abstract

During locomotion, cervical muscles must be active to stabilize the head as the body accelerates and decelerates. We hypothesized that cervical muscles are also part of the linked chain of axial muscles that provide core stabilization against torques applied to the hip joint by the extrinsic muscles of the legs. To test whether specific cervical muscles play a role in postural stabilization of the head and/or core stabilization of the pelvic girdle, we used surface electromyography to measure changes in muscle activity in response to force manipulations during constant speed running and maximum effort counter-movement jumps. We found that doubling the mass of the head during both running and maximum effort jumping had little or no effect on (1) acceleration of the body and (2) cervical muscle activity. Application of horizontal forward and rearward directed forces at the pelvis during running tripled mean fore and aft accelerations, thereby increasing both the pitching moments on the head and flexion and extension torques applied to the hip. These manipulations primarily resulted in increases in cervical muscle activity that is appropriate for core stabilization of the pelvis. Additionally, when subjects jumped maximally with an applied downward directed force that reduced acceleration and therefore need for cervical muscles to stabilize the head, cervical muscle activity did not decrease. These results suggest that during locomotion, rather than acting to stabilize the head against the effects of inertia, the superficial muscles of the neck monitored in this study help to stabilize the pelvis against torques imposed by the extrinsic muscles of the legs at the hip joint. We suggest that a division of labor may exist between deep cervical muscles that presumably provide postural stabilization of the head versus superficial cervical muscles that provide core stabilization against torques applied to the pelvic and pectoral girdles by the extrinsic appendicular muscles.

## Abstract o (Spanish)

Durante la locomoción, los músculos cervicales deben estar activos para estabilizar la cabeza a medida que el cuerpo acelera y desacelera. Presumimos que los músculos cervicales también son parte de la cadena unida de músculos axiales que brindan estabilización central contra las torsiones aplicadas a la articulación de la cadera por los músculos extrínsecos de las piernas. Para evaluar si los músculos cervicales específicos desempeñan un papel en la estabilización postural de la cabeza y/o la estabilización central de la cintura pélvica, utilizamos electromiografía de superficie para medir los cambios en la actividad muscular en respuesta a las manipulaciones de fuerza durante la carrera a velocidad constante y los saltos con contramovimiento de esfuerzo máximo. Descubrimos que duplicar la masa de la cabeza durante la carrera y el salto de máximo esfuerzo tuvo poco o ningún efecto sobre (1) la aceleración del cuerpo y (2) la actividad de los músculos cervicales. La aplicación de fuerzas horizontales dirigidas hacia adelante y hacia atrás en la pelvis durante la carrera triplicó las aceleraciones medias hacia adelante y hacia atrás, aumentando así tanto los momentos de cabeceo en la cabeza como los pares de flexión y extensión aplicados a la cadera. Estas manipulaciones dieron como resultado principalmente aumentos en la actividad de los músculos cervicales que son apropiados para la estabilización central de la pelvis. Además, cuando los sujetos saltaban al máximo aplicando una fuerza dirigida hacia abajo que reducía la aceleración y, por lo tanto, la necesidad de los músculos cervicales para estabilizar la cabeza, la actividad de los músculos cervicales no disminuía. Sugerimos que puede existir una división del trabajo entre los músculos cervicales profundos que presumiblemente brindan estabilización postural de la cabeza versus los músculos cervicales superficiales que brindan estabilización central contra los torques aplicados a las cinturas pélvica y pectoral por los músculos apendiculares extrínsecos.

## Abstrakt (German)

Während der Fortbewegung müssen die Halsmuskeln aktiv sein, um den Kopf zu stabilisieren, wenn der Körper beschleunigt und abbremst. Wir stellten die Hypothese auf, dass die zervikalen Muskeln auch Teil der verknüpften Kette axialer Muskeln sind, die eine Rumpfstabilisierung gegen Drehmomente bereitstellen, die von den äußeren Muskeln der Beine auf das Hüftgelenk ausgeübt werden. Um zu testen, ob bestimmte Halsmuskeln eine Rolle bei der Haltungsstabilisierung des Kopfes und/oder der Rumpfstabilisierung des Beckengürtels spielen, verwendeten wir Oberflächen-Elektromyographie, um Veränderungen der Muskelaktivität als Reaktion auf Kraftmanipulationen während des Laufens mit konstanter Geschwindigkeit und der Gegenbewegung mit maximaler Anstrengung zu messen springt. Wir fanden heraus, dass die Verdopplung der Kopfmasse sowohl beim Laufen als auch beim Springen mit maximaler Anstrengung wenig oder gar keinen Einfluss auf (1) die Beschleunigung des Körpers und (2) die Aktivität der Halsmuskeln hatte. Die Anwendung von horizontal nach vorne und hinten gerichteten Kräften auf das Becken während des Laufens verdreifachte die mittleren Beschleunigungen nach vorne und hinten, wodurch sowohl die Nickmomente auf den Kopf als auch die auf die Hüfte ausgeübten Flexions- und Extensionsdrehmomente erhöht wurden. Diese Manipulationen führten in erster Linie zu einer Erhöhung der zervikalen Muskelaktivität, die für die Rumpfstabilisierung des Beckens geeignet ist. Wenn die Probanden mit einer nach unten gerichteten Kraft maximal sprangen, die die Beschleunigung reduzierte und daher die Halsmuskeln zur Stabilisierung des Kopfes benötigt wurden, nahm die Aktivität der Halsmuskeln nicht ab. Diese Ergebnisse legen nahe, dass die in dieser Studie überwachten oberflächlichen Halsmuskeln während der Fortbewegung dazu beitragen, das Becken gegen Drehmomente zu stabilisieren, die von den äußeren Beinmuskeln am Hüftgelenk ausgeübt werden, anstatt den Kopf gegen die Auswirkungen der Trägheit zu stabilisieren. Wir schlagen vor, dass eine Arbeitsteilung zwischen tiefen Halsmuskeln bestehen kann, die vermutlich für die posturale Stabilisierung des Kopfes sorgen, und oberflächlichen Halsmuskeln, die eine Kernstabilisierung gegen Drehmomente bieten, die von den extrinsischen Blinddarmmuskeln auf den Becken- und Brustgürtel ausgeübt werden.

## Abstrait (French)

Pendant la locomotion, les muscles cervicaux doivent être actifs pour stabiliser la tête lorsque le corps accélère et décélère. Nous avons émis l“hypothèse que les muscles cervicaux font également partie de la chaîne liée des muscles axiaux qui assurent la stabilisation du noyau contre les couples appliqués à l”articulation de la hanche par les muscles extrinsèques des jambes. Pour tester si des muscles cervicaux spécifiques jouent un rôle dans la stabilisation posturale de la tête et/ou la stabilisation centrale de la ceinture pelvienne, nous avons utilisé l“électromyographie de surface pour mesurer les changements dans l”activité musculaire en réponse aux manipulations de force pendant la course à vitesse constante et les sauts de contre-mouvement à effort maximal. Nous avons constaté que doubler la masse de la tête pendant la course et le saut à effort maximal avait peu ou pas d“effet sur (1) l”accélération du corps et (2) l“activité des muscles cervicaux. L”application de forces horizontales dirigées vers l“avant et vers l”arrière au niveau du bassin pendant la course a triplé les accélérations moyennes vers l“avant et vers l”arrière, augmentant ainsi à la fois les moments de tangage sur la tête et les couples de flexion et d“extension appliqués à la hanche. Ces manipulations ont principalement entraîné une augmentation de l”activité des muscles cervicaux, ce qui est approprié pour la stabilisation centrale du bassin. De plus, lorsque les sujets sautaient au maximum avec une force dirigée vers le bas qui réduisait l“accélération et donc le besoin de muscles cervicaux pour stabiliser la tête, l”activité des muscles cervicaux ne diminuait pas. Ces résultats suggèrent que lors de la locomotion, plutôt que d“agir pour stabiliser la tête contre les effets de l”inertie, les muscles superficiels du cou suivis dans cette étude aident à stabiliser le bassin contre les couples imposés par les muscles extrinsèques des jambes au niveau de l“articulation de la hanche. Nous suggérons qu”une division du travail peut exister entre les muscles cervicaux profonds qui assurent vraisemblablement la stabilisation posturale de la tête et les muscles cervicaux superficiels qui assurent la stabilisation centrale contre les couples appliqués aux ceintures pelvienne et pectorale par les muscles appendiculaires extrinsèques.

## 抽象的 (Japanese)

移動中は、体が加速および減速するときに頭を安定させるために頸部の筋肉がアクティブになっている必要があります。頸部の筋肉は、脚の外因性の筋肉によって股関節に加えられるトルクに対してコアの安定化を提供する軸方向の筋肉のリンクされたチェーンの一部でもあると仮定しました。特定の頸部の筋肉が頭の姿勢の安定化および/または骨盤ガードルのコアの安定化に役割を果たすかどうかをテストするために、表面筋電図を使用して、定速走行および最大努力の反動中の力の操作に応じた筋肉活動の変化を測定しましたジャンプします。ランニングと最大エフォートジャンプの両方で頭の質量を2倍にすると、(1)体の加速と(2)頸部の筋肉の活動にほとんどまたはまったく影響がないことがわかりました。3倍の平均前後加速度を実行しているときに、骨盤に水平方向の前方および後方に向けられた力を加えると、頭のピッチングモーメントと、股関節にかかる屈曲および伸展トルクの両方が増加します。これらの操作は、主に骨盤のコアの安定化に適した頸部の筋肉活動の増加をもたらしました。さらに、被験者が下向きの力を加えて最大にジャンプした場合、加速度が低下し、したがって頸部の筋肉が頭を安定させる必要がありましたが、頸部の筋肉の活動は低下しませんでした。これらの結果は、運動中に、慣性の影響に対して頭を安定させるように作用するのではなく、この研究で監視された首の表在筋が、股関節の脚の外因性筋肉によって加えられるトルクに対して骨盤を安定させるのに役立つことを示唆している。おそらく頭の姿勢の安定化を提供する深頸部の筋肉と、外因性の四肢骨格によって骨盤および胸帯に加えられるトルクに対するコアの安定化を提供する浅頸部の筋肉との間に分業が存在する可能性があることを示唆します。

## Introduction

**Fig. 1:**
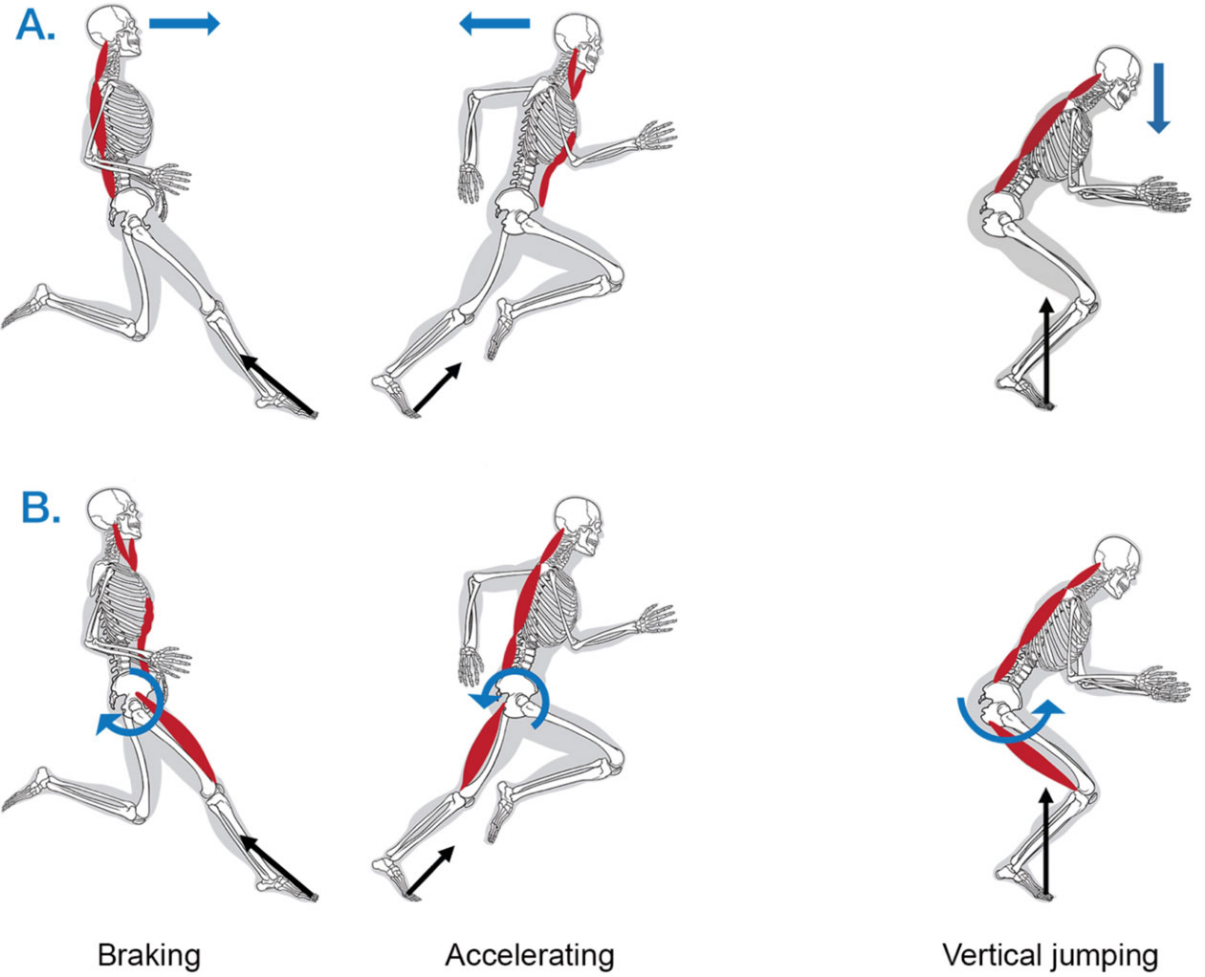
Illustration of the hypothesized functions of the muscles of the neck during accelerating and decelerating steps and vertical jumping. A. To control posture of the head in response to the tendency of body segments to be left behind during accelerations due to inertia (blue arrows) during running the posterior muscles of the neck must be active during forward braking steps whereas the anterior muscles of the neck must be active during forward accelerating steps. B. To provide core stabilization of the pelvic girdle during running, we hypothesize that the anterior muscles of the neck are part of a linked chain of activation of hypaxial muscles that resist the torques applied at the hip joint by the limb protractor muscles (blue arrow) during braking steps. Similarly, posterior muscles of the neck are hypothesized to be part of linked chain of activation of epaxial muscles that resist the torques applied at the hip joint by the limb retractor muscles (blue arrow) during accelerating steps. Note that, during running, muscle activity necessary for postural stabilization of the head conflicts with the hypothesized activity necessary for core stabilization. A. To control posture of the head in response to the inertia of the head as the body accelerates (blue arrow) during jumping, the posterior muscles of the neck must be active. B. To provide core stabilization during jumping, we hypothesize that posterior muscles of the neck are part of a linked chain of activation of epaxial muscles that resist the torques applied at the hip joint by the limb retractor muscles (blue arrow) during acceleration.

In coordinated movement, active muscles serve two functions: stabilization of joints against forces that would tend to provide unwanted displacement of those joints or the production of positive or negative work at a joint. During locomotion, although the axial muscles of humans produce very little or no work, first principles indicate that they must be active to stabilize the axial skeleton against three different forces. First, as the body accelerates and decelerates in a walking, running, or jumping step, axial muscles must exert force to provide **postural stabilization** (Fig. 1A) of joints of the axial skeleton against the tendency of body segments (e.g., head and trunk) to be “left behind” due to their inertia (Thorstensson et al., 1982; Fife et al., 2001; Goodman, 2004; Schilling and Carrier, 2010; Schilling, 2011; Yegian et al., 2021). Second, to provide a fixed skeletal foundation from which the extrinsic appendicular muscles can apply torques to the legs about the hip joint, axial muscles must exert force to ensure **core stabilization** (Fig. 1B) of that skeletal foundation (i.e., pectoral and pelvic girdles and axial skeleton) (Carrier, 1993; Fife et al., 2001; Goodman, 2004; Kibler et al., 2006; Schilling and Carrier, 2009, 2010; Schilling, 2011). Third, Earth's gravity represents a third force that requires activity of axial muscles for postural stabilization (Schilling, 2011). However, in most circumstances, gravitational moments about human axial joints are relatively small as is illustrated by the very low levels of recorded muscle activity when humans stand still.

What role, if any, does the human neck play in postural and core stabilization? Certainly, controlling posture of the head on the trunk requires active cervical muscles to resist the tendency of the head to be left behind as the body accelerates and decelerates (Fig. 1A). In contrast to our closest relatives, the other great apes, humans are habitual bipeds who do not walk and run on their forelimbs. Thus, we can reasonably assume that the human neck does not need to provide significant core stabilization for the extrinsic muscles of forelimb and pectoral girdle during locomotion. However, we suspect that the musculoskeletal system of the neck and jaw contribute to core stabilization of the pelvic girdle during locomotion (Fig. 1B). We hypothesize that the axial muscles of the abdomen, thorax, neck, and jaw are parts of a linked chain that must be activated in an integrated fashion whenever the extrinsic muscles of the leg apply significant moments at the hip joint. From the perspective of a linked system, it is not difficult to imagine how the neck musculoskeletal system would be important in core stabilization of the pelvis during vigorous accelerations. If some cervical muscles do participate in core stabilization during locomotion, motor control may require a balanced coactivation of cervical antagonists to meet sometimes-conflicting demands of core stabilization versus postural stabilization (Fig. 1A, B).

The musculoskeletal core is most often defined as the lumbo-pelvic hip complex, consisting of the lumbar spine, pelvis, and hip joints, and the active and passive tissues that produce or restrict motion of these segments (Willson et al., 2005). As stated above, we address the possibility that the core extends above the lumbar spine and abdomen all the way to the head. A definition that is closer to our expectations defines the musculoskeletal core “as the axial skeleton (which includes the pelvic and shoulder girdles) and all soft tissues (i.e., articular and fibro-cartilage, ligaments, tendons, muscles, and fascia) with a proximal attachment originating on the axial skeleton, regardless of whether the soft tissue terminates on the axial or appendicular skeleton (upper and lower extremities)” (Behm et al., 2010).

We had two goals in conducting this study. Using surface electromyography, we sought to (1) document the activity patterns of a set of superficial cervical muscles during steady state running and the acceleration phase of counter movement jumping; and (2) test the functional role of these muscles. To test the muscular function, we manipulated the physical conditions in which the subjects performed to either alter the forces cervical muscles must generate to maintain postural stabilization of the head, or to increase the flexion and extension torques that the extrinsic limb muscles apply at the hip joint during running. To increase the forces associated with postural stabilization, we added mass to the head, roughly doubling its inertia. To increase the flexion and extension torques applied at the hip joint during steady state running we applied large horizontally directed rearward and forward forces to a waist harness worn by the subjects. To reduce the forces associated with postural stabilization during counter movement jumping we applied a vertically directed (downward) force to a waist harness worn by the subjects, which reduced the acceleration of the body. These manipulations lead to distinct predictions of changes in muscle activation for the two hypotheses of muscle function (Fig. 1). Additionally, during running, because the extensor moments at the hip are larger during the first than second half of the stance phase and conversely flexor moments at hip are larger during the second half of stance (Winter, 1983; Roberts & Belliveau, 2005; Hamner et al., 2010; Bezodis et al., 2014), specific predictions can be made regarding the phase relationships of increased muscle activity that would support the core stabilization hypothesis. Specifically, the core stabilization hypothesis leads to the expectations that cervical epaxial muscles will exhibit a pronounced increase in activity during the first half of the step in the rearward pull trials and activity of the cervical hypaxial muscles will significantly increase during the second half of the step in the forward pull trials.

## Methods

### Study participants

Sixteen healthy males (body mass: 73.7 ± 7.3 kg, mean ± SD; age: 26 ± 3 years) participated in this study. The study was restricted to males because males have cervical muscles of substantially larger cross section area than females (Vasavada et al., 2001), providing larger fields for surface electrodes, therefore yielding recordings that are less likely to be impacted by cross-talk from adjacent muscles. The focus of this study is on human locomotor performance so results gathered here should also apply to human females. Potential subjects were excluded from the study if they acknowledged head, neck, or back pain. To limit the effect of fatigue during the experiment, only subjects who were physically active and either regular distance runners or regular participants in other cardiovascular sports were recruited as subjects. All gave their informed consent to voluntarily participate in this study, which was approved by the IRB Committee of the University of Utah (IRB 00108020).

### Monitoring of acceleration

Acceleration during running trials was measured with a 3-axis, 4 g accelerometer (SEN-12756, Spark Fun, Boulder, CO) mounted on the left lateral side of the neck midway between the mastoid process and clavicle. We were most interested in the acceleration experienced by the head. However, the necessity of repeatedly putting on and removing the headgear made consistent orientation of an accelerometer on the head impossible. For this reason, we measured acceleration with a three-axes accelerometer attached to the neck. Although there may be differences in acceleration of the head and neck during running, these are small compared to the vertical and horizontal acceleration experience by the entire body. The accelerometer was positioned such that the x-axis was orthogonal to gravity when the subject was standing upright. The accelerometer was attached to the neck with the CoFlex Tape (Andover, Monroe, LA) that completely covered the neck to reduce movement artifact. Acceleration during maximal effort countermovement jumps (i.e., a jump in which the subject rapidly squats and then immediately jumps upward) was monitored with a force plate (Kistler, 9281B SN, Winterthur, Switzerland). We used the ground reaction force time series to calculate the acceleration of the subject's center of mass according to the methods of Linthorne (2001). These signals were sampled at 2000·Hz and stored in digital form on an Apple Macintosh computer.

### Electromyography

We used surface electromyography to examine the activation of seven cervical muscles and one jaw adductor muscle (muscles and electrode positions are detailed in Table 1). Note, as described in Table 1, potential for cross-talk from adjacent muscles exists for some of the electrode placements. Disposable Ag-AgCl electrodes (H124SG, Covidien Kendall, Minneapolis MN) with a circular uptake area of 1.6 cm diameter and an inter-electrode distance of 2.5 cm were applied to muscles on the right side of the neck with the exception of the semispinalis which was monitored on both right and left sides. All electrodes were carefully secured by wrapping the neck with co-flex wrap to minimize movement artefacts. If any electrodes shifted from the placement site or displayed uncharacteristic background noise we discarded all recordings from the bad electrode for that subject. Electromyographic signals were filtered above 1000 Hz and below 100 Hz with Grass P511 AC amplifiers. Note that our high-pass filter setting of 100 Hz is well above recommendations (Van Boxtel, 2001). See the Limitations of the Study section below for a discussion of the implications of this. The EMG signals were amplified approximately 2000 times, sampled at 2000·Hz, and stored in digital form on an Apple Macintosh computer.

**Table 1:** Description of muscles studied

Muscle	Hypothesized function during locomotion	Description of electrode placement	Sources of possible cross-talk
Sternohyoid	Stabilization against extension moments	Mid-neck, 1.5 cm lateral from midline (C 4)	Platysma
Sternocleidomastoid	Stabilization against extension moments	One third of muscle length below insertion (Sommerich et al., 2000)	None
Masseter	Stabilization against extension moments	Anterior aspect, between zygomatic arch and mandible (Castroflorio et al., 2005)	None
Levator scapulae	Stabilization against flexion moments	Between the posterior margin of the sternocleidomastoideus muscle and the anterior margin or the trapezius (Queisser et al., 1994; Schuldt and Harms-Ringdahl 1988; Sommerich et al., 2000)	Trapezius and middle scalene
Upper trapezius	Stabilization against flexion moments	Halfway along a line drawn between the spine of the 7th cervical vertebra and the acromion (Jensen et al., 1993; Mercer, 2002)	None
Splenius capitis	Stabilization against flexion moments	6–8 cm laterally of the median line at the level of C4 (Keshner et al. 1989; Queisser et al., 1994).	None
Semispinalis capitis (right)	Stabilization against flexion moments	2 cm below the occipital bone at the level of C1/C2 and 2 cm lateral of the median line (Keshner et al., 1989; Queisser et al., 1994; Sommerich et al., 2000)	Upper Trapezius, pars descendens
Semispinalis capitis (left)	Stabilization against flexion moments	2 cm below the occipital bone at the level of C1/C2 and 2 cm lateral of the median line (Keshner et al., 1989; Queisser et al., 1994; Sommerich et al., 2000)	Upper Trapezius, pars descendens

### Protocol

To reduce variability in our data with respect to cervical muscle activity we asked subjects to avoid any unnecessary head movements (e.g., nodding or looking sideways), and to avoid speaking and laughing while data were being collected. Prior to data collection subjects were able to practice running and jumping with all manipulations until they felt comfortable continuing with the tests.

*Running trials—*Subjects ran at constant speed on a treadmill set to 2.7 ms^–1^. An accelerometer was placed on the subject's right foot to allow synchronization of the stride cycle with the EMG recordings. During a warmup period, we recorded the preferred stride frequency of the subject and set a metronome which the subject matched in all subsequent running trials to ensure that the stride frequency remained consistent. Synchronization between footfall and the metronome was easily observed by the experimenters and if a trial contained any non-synchronized steps the entire trial was immediately discarded. To increase the flexion and extension torques applied at the hip joint in the running trials we applied large (20–27% BWt) forward and rearward directed horizontal forces to the subject via elastic bands attached to a waist harness throughout the duration of the trial (Chang and Kram, 1999). The elastic bands were attached to the harness laterally over the right and left hip joints. These trials were rejected if the applied force dropped below 150 N or exceeded 200 N during the trial. To increase the mass of the head, subjects wore a padded head harness with 2.25 kg mass added to the right and left sides of the head harness (4.5 kg total), roughly doubling the mass of the head. The snug fitting and adjustable harness held the added masses snuggly to the head and close to the vertical position of the center of mass of the head. Consequently, for this experiment, we assumed that the added mass roughly doubled rotational moments due to the inertia of the head. In each trial, after the subject reached a consistent velocity and rhythmic stride, in phase with the metronome, we recorded data for 20 locomotor cycles. To prevent fatigue, each trial lasted approximately 20–25 s and subjects rested for 2 to 3 min between trials. No subjects reported fatigue during the data collection. We collected two trials for each manipulation and analyzed data from the second trial unless there was a problem such as an unconnected electrode or accelerometer in which case the first trial was analyzed.

The analysis would have been strengthened by simultaneous monitoring of ground forces so that calculations of the moments at the hip could have been included. Unfortunately, the equipment available for this study did not allow this. Nevertheless, as discussed below (see Limitations), previous studies have quantified moments at the hip joint during running at constant speed and when hip moments are manipulated through incline running. These studies provide confidence that horizontal force manipulations used in this study changed the muscle moments at the hip joint in the intended direction. Additionally, these published hip joint moments lead to specific predictions regarding axial muscle activity to provide core stabilization of the pelvis during constant speed running.

*Jumping trials*—Study participants were instructed to stand on the force plate while a baseline measurement was taken and then jump as high as possible in a countermovement jump with their arms at held at their sides or with their hands resting comfortably on their hips. Each subject completed four control jumps (supplemental video 1), four added gravity jumps (supplemental video 2), and four added head-mass jumps (supplemental video 3) in random order and with rest time in between each jump. The increase in vertical force (approximately 270 N standing, 230 N bottom of counter movement, 280 N toe off) for the added gravity jumps was applied by stretched elastic bands with lines that were directed vertically with pullies positioned directly below the subject's hips when they stood on the force plate. To increase the mass of the head, subjects wore a head harness with 2.25 kg mass added to the right and left sides of the harness. The configuration of waist and head harness are shown in Supplemental Videos 2 and 3, respectively. After the 12 maximal effort jumps, all participants were instructed to perform a series of smaller effort jumps aiming for 10, 25, 50, 75, 90% of maximal effort on the force plate. Subjects completed 4 jumps at each of these lower effort levels in random order with rest time in between each jump.

### Analysis of acceleration

Analysis of the acceleration from the running trials began by smoothing each trace using a rolling mean over a period of 10 ms. For control trials and the added head mass trials, the baseline for the vertical acceleration traces was found by averaging all the minimum values, which represented the periods during the strides when the subjects were in flight phase. This offset voltage was subtracted from each data point. Peak positive and negative values were recorded for each running step. The offset (i.e, zero acceleration) voltage for the horizontal accelerometer was determined by calculating the mean voltage of an integral number of locomotor cycles. The offset voltage was subtracted from each data point and peak positive and negative values were recorded for each running step. Accelerometers were calibrated by positioning each axis vertically to allow measurement of the voltage associated with the acceleration of gravity.

The amplitude of the applied horizontal forces proved large enough to eliminate all forward acceleration by the subjects during the forward pull trials and all forward deceleration by the subjects during rearward pull trials. In other words, the applied horizontal forces were large enough that the runners provided no forward braking forces to the treadmill when they were pulled backward and no forward accelerating forces to the treadmill when they were pull forward. In fact, the mean acceleration during the entire step when subjects were pulled either forward or backward (Table 4) was almost three-fold greater than the mean acceleration during the first and second halves (respectively) of the step in the control trials. We therefore assumed that the baseline for the horizontal acceleration during the forward pull trials was the mean of all peak voltages during each step and the baseline during the rearward pull trials was the mean of the minimum values during each step. Note that we did not correct for the change in orientation of the accelerometers due to the subjects leaning forward during the rearward pull trials and leaning backward during the forward pull trials. From video recordings, the change in orientation of the trunk and neck ranged from + 8 to + 28 degrees when subjects were pulled forward and from –18 to –24 degrees when they were pulled backwards. These changes in orientation of the accelerometer placed on the neck resulted in a minor underestimation (less than 12%, from cos28^o^ = adjacent/hypothesis = 0.883) of the actual fore and aft accelerations induced by the application of horizontal forces to the running subjects. Given that the mean forward acceleration during the entire step when subjects were pulled backwards (Table 4) was almost three-fold greater than the mean acceleration during the second half of the step in the control trials, a potential 12% underestimation is acceptable. Based on visual examination of slow-motion video recordings, the position of the head relative to the shoulders remained steady (i.e., the head did not wobble) during all trials.

Acceleration during maximal effort countermovement jumps was monitored with a force plate (Kistler, 9281B SN, Winterthur, Switzerland). We used the impulse momentum method outlined by Linthorne to integrate the area under the force—time curve for the period of the jump to calculate the acceleration of the subjects’ center of mass (2001).

### Analysis of EMG

To examine the pattern of muscle activity during steady speed running, we analyzed the EMGs for each muscle from 20 locomotor cycles. Locomotor cycles were defined based on the foot strike signal from the accelerometer placed on the dorsal surface of the right foot. Thus, the sampling window for the locomotor cycle began and ended with touch-down of the right foot. To examine the pattern of muscle activity during countermovement jumping, the sampling window began the moment the vertical force trace dropped below 10% of body weight at the initiation of the countermovement. The sampling window contained the entire push-off phase of the jump including the time in the air and ended when the force trace indicated initiation of landing by rising to 10% of body weight. Before analysis, the average rectified background voltage when the muscles were relaxed was subtracted from the signal. This background signal was measured while subjects were lying prone or supine with their head supported. The lowest 500 data points were selected and averaged.

### Statistics

For both the running and countermovement jump trials, we treated each force manipulation as separate experiments: running with added head mass (Table 5), with added forward-directed horizontal force, and with added rearward-directed horizontal force (Table 6), and jumping with added head mass, and with added downward directed force (Table 7). In each case, amplitude of the EMG (e.g., integrated, maximum, and mean voltages) recorded during a given force manipulation was compared to that recorded during the appropriate control trials with the non-parametric, Wilcoxon signed-rank test (paired, one-tailed) using a R data analysis package (Wickham et al., 2019). We assumed the results were significantly different when the *P*-value was less than 0.05 after applying the Holm–Bonferroni Sequential Correction (Holm, 1979) for the eight muscles to control the error rate (Type I errors). This pairwise approach than a multivariate approach such as a Linear Mixed Model, because each force manipulation was an independent variable, applied in separate trials which eliminated potential interactions with other force manipulations. Additionally, because the EMG data were not normally distributed (Shapiro–Wilk test for normality), use of a repeated-measure ANOVA, MANOVA, or LMM was not possible.

The exception to the comparison of force manipulation to control trials were the trials in which added head mass and forward- or rearward-directed force was added simultaneously to running subjects (Table 6). These trials were done to control for the change in running posture produced by the horizontal forces. The forward lean when the subjects ran with a rearward-directed force and backward lean when subjects ran with a forward-directed force raised the possibility that the change in activity observed in the cervical muscles could have resulted from an increased need for postural stabilization rather than the intended elevated demand for core stabilization due to increased extensor or flexor moments at the hip joint. In this case, the appropriate pairwise comparison was to the forward or rearward horizontal force trials rather than to the control trials.

To allow comparison of control to force manipulation trials, the rectified EMGs were integrated and averaged for each subject. To enable averaging across multiple subjects we normalized the values to the average maximum voltages measured during maximum voluntary contractions (MVC) trials (N = 4) for each subject. For the MVC trials, muscle activity was recorded as a subject pushed maximally against the experimenter's extended arms as the subject lay horizontally in supine (neck flexion) and prone (neck extension) postures. Because we were manipulating forces of the head in the fore-aft direction, and moments of the hip oriented in the same direction, we felt it was more appropriate to measure MVC in fore-aft motions of the neck.

To provide graphic illustrations of the effects of the force manipulations, each normalized EMG sample was divided into 100 bins for the running strides and 200 bins for the counter movement jumps. The average of the voltage was calculated for each bin (Carrier, Deban, & Fischbein, 2006). Averages for each subject, for each bin, were calculated for the 20 locomotor cycles and the 4 jump trials. Then, averages and 95% confidence intervals for each bin were calculated for the 16 subjects.

## Results

### Effect of force manipulations on acceleration of the body

Doubling the mass of the head during steady state running did not change mean vertical, fore, and aft acceleration of the subjects relative to control trials (Table 4). Although the added horizontal forward- and rearward-directed forces did not change the mean vertical acceleration, these forces roughly tripled the mean fore and aft accelerations relative to control trials. Doubling the mass of the head during the added forward- and rearward-directed force trials did not change the accelerations with the one exception of a 7.3% drop in mean vertical acceleration with added head mass in the added rearward-directed force trials. Comparisons of the fore and aft accelerations indicate that we succeeded in inducing roughly equal increases in acceleration in the two directions (Table 4).

During maximum effort countermovement jumps, application of a downward directed vertical force of approximately 270 N reduced the force impulse (above body weight) applied by the subjects to the force plate from an average of 203.7 ± 22.2 N s to 159.5 ± 23.4 N s; a 21.7% reduction. In contrast, doubling the inertia of a subject's head did not change the force impulse, 203.7 ± 22.2 N s versus 210.5 ± 19.1 N s, respectively. Thus, we successfully reduced acceleration of the body when we added the downward force but did not change acceleration when we added mass to the head.

### Cervical muscle activity during running at a sustainable speed

**Fig. 2:**
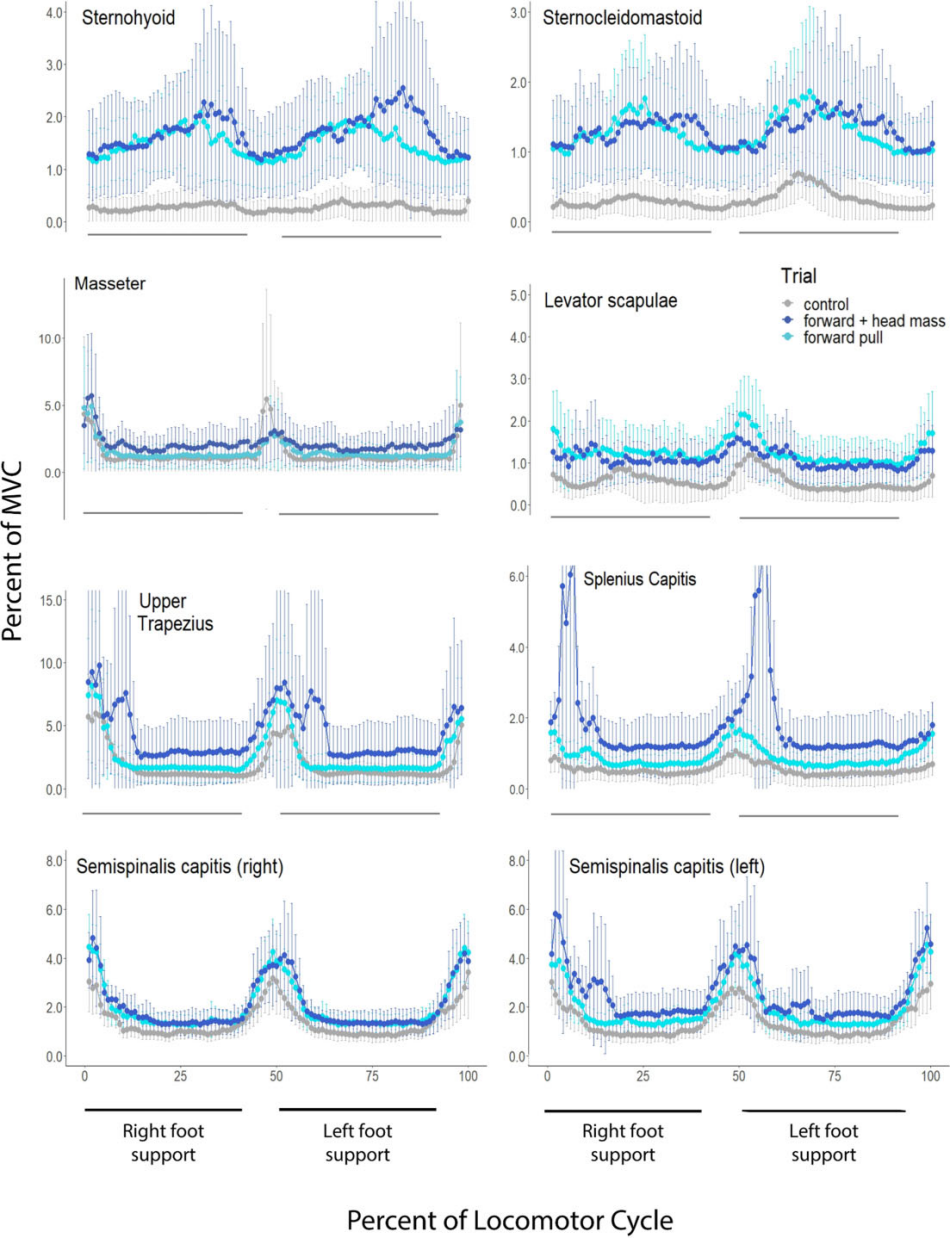
Plots of average muscle activity of subjects during running comparing trials in which the subjects ran while resisting a force that acted to pull them forward on the treadmill resulting in increased braking forces on the substrate and increased flexion torques applied at the hip joint (light blue line) to control trial (gray line). Also shown is the average muscle activity in trials in which subjects resisted the forward force and wore a weighted head harness that roughly doubled the mass of their heads (dark blue line). All muscles are on the right side of the neck unless noted otherwise. Values for the muscle activity are expressed as a mean % of MVC. Error bars represent 95% confidence intervals.

**Fig. 3:**
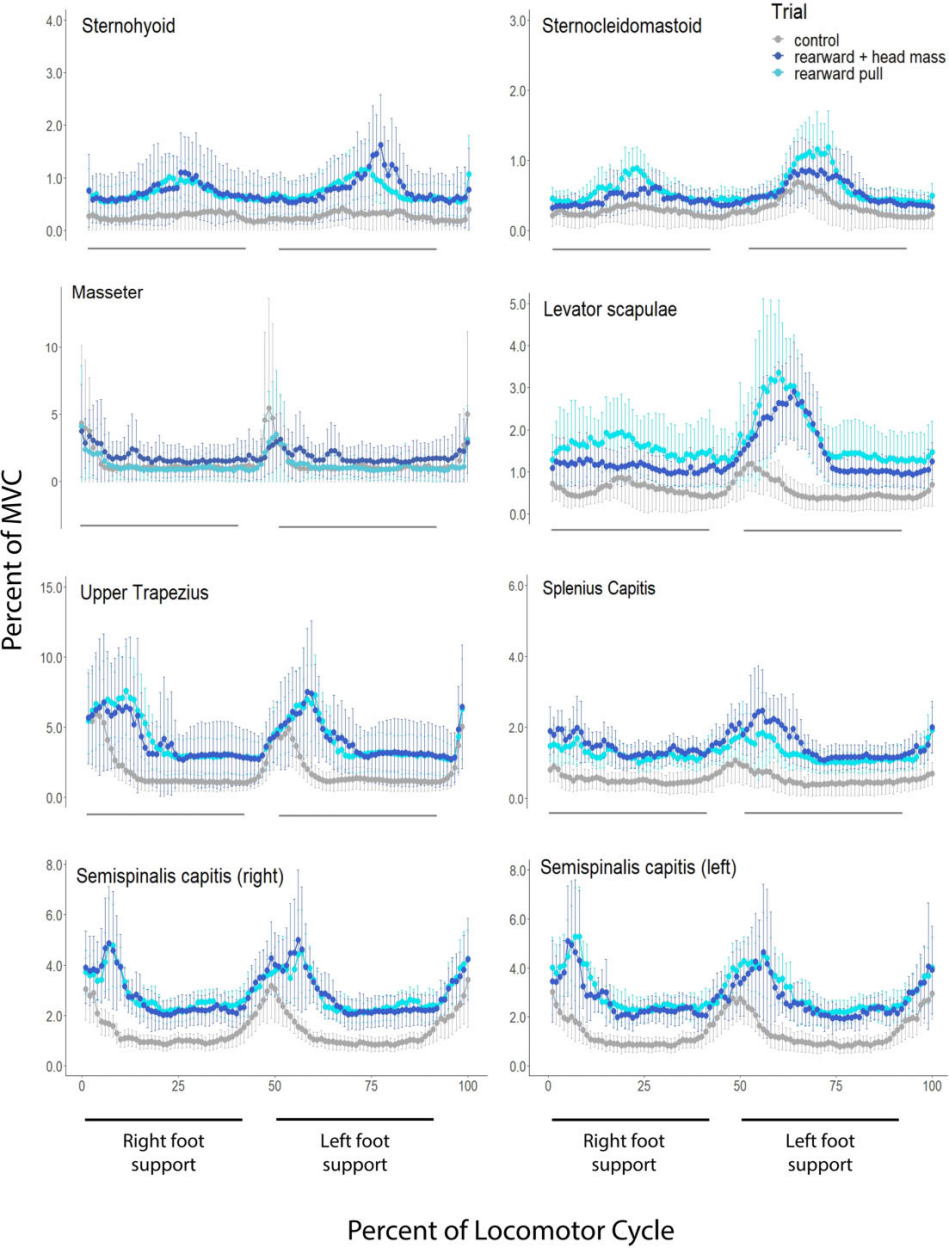
Plots of average muscle activity of subjects during running comparing trials in which the subjects ran while resisting a force that acted to pull them backwards on the treadmill resulting in increased accelerating forces on the substrate and increased extension torques applied at the hip joint (light blue line) to control trials (gray line). Also shown, is the average muscle activity in trials in which subjects resisted the rearward force and wore a weighted head harness that roughly doubled the mass of their heads (dark blue line). All muscles are on the right side of the neck unless noted otherwise. Values for the muscle activity are expressed as a mean % of MVC. Error bars represent 95% confidence intervals.

In the cervical muscles examined in this study, maximum and average activity during running unencumbered, at a sustainable speed, was of a relatively low level compared to maximum voluntary contraction, 0.4–6.0% (Figs. 2 and 3, Supplementary Fig. 1). Broadly, the activity was characterized by (1) a low-level tonic activity of 0.2 to 2.0% of MVC, and (2) low biphasic activity synchronized with the stride. In some subjects, the biphasic activity was not apparent in the masseter and sternohyoid muscles. The biphasic pulses of the sternohyoid and sternocleidomastoid occurred during mid-support, and in the case of the sternocleidomastoid peak activity occurred during contralateral support. Biphasic activity of the levator scapulae began slightly before foot contact with peak activity occurring slightly after contact. As was true for the sternocleidomastoid, phasic activity associated with contralateral foot fall was of higher amplitude than that associated with ipsilateral foot fall. The trapezius, splenius capitis, semispinalis capitis muscles exhibited peak activity prior to foot contact with roughly equal amplitude phasic bursts associated with ipsi- and contralateral steps.

To test the hypothesis that superficial cervical muscles are active to control the posture of the head during a running stride (Fig. 1A) we increased (roughly doubled) the mass of the head and compared activity in the added mass trials to that of control trials. Note that running speed and stride frequency were held constant and the added mass did not change the mean vertical and fore-and-aft accelerations of the body, measured at the neck (Table 4). Thus, we can be confident that this manipulation roughly doubled the forces cervical muscles had to produce to provide postural stabilization of the head. Results are summarized in Table 5; expressed as a ratio of activity during the manipulation over the activity during control trials. The added head mass did not significantly affect muscle activity seen in the sternohyoid, sternocleidomastoid, splenius capitis, nor the semispinalis capitis muscles. Activity in the levator scapulae and the upper trapezius muscles increased slightly, 21 to 26%, respectively, and the activity in the masseter decreased 29% compared to control trials. Plots of average activity for each of the 16 subjects for the Sternohyoid (Sup. Fig. 2) and Semispinalis (Sup. Fig. 3) muscles show that in some subjects the added head mass trial was virtually undistinguishable from the control trial. In other subjects, the added mass trial did exhibit higher muscle activity, whereas in a few subjects muscle activity during the added mass trial was less than the control trial. Although a larger subject sample might have revealed significant increases in muscle activity in those muscles that did not exhibit a significant change, the results of this experiment are consistent with the possibility that the superficial cervical muscles monitored in this study do not play a large role in postural stabilization of the head during running.

To test the hypothesis that superficial cervical muscles are active to provide core stabilization during a running stride (Fig. 1B) we added forward and rearward directed forces and compared activity to that of control trials. We observed that activity of the monitored muscles increased during both the forward and rearward added horizontal force trials relative to control trials (Figs. 2 and 3, Table 6). The exceptions were the masseter muscle and the upper trapezius muscle during the forward pulling trials. Importantly, for the sternohyoid and sternocleidomastoid muscles the increased activity was significantly greater during trials in which forward directed forces were applied (Fig. 2, Table 6). Conversely, for the upper trapezius, splenius capitis, and semispinalis capitis muscles the increased activity was significantly greater in the trials in which rearward-directed force were applied (Fig. 3, Table 6). These results are consistent with the increase activity being associated with core stabilization rather than postural stabilization of the head (Table 2).

**Table 2:** Predictions for running experiments

Experiment	Force manipulation	Prediction of postural stabilization hypothesis	Prediction of core stabilization hypothesis
**Added head mass**	Increased forces cervical muscles must produce to maintain postural stabilization	**Increase in activity** of cervical muscles relative to control trials	**No change in activity** of cervical muscles relative to control trials
**Added forward-directed force**	Increased flexion moments applied to hip joint	**No change in activity** of cervical muscles relative to control trials	**Increase in activity** of cervical hypaxial muscles relative to control trials
**Added rearward-directed force**	Increased extension moments applied to hip joint	**No change in activity** of cervical muscles relative to control trials	**Increase in activity** of cervical epaxial muscles relative to control trials
**Added head mass and forward-directed force**	Increased forces cervical muscles must produce to maintain postural stabilization in forward-directed force trials	**Increase in activity** of cervical muscles relative to forward-directed pull trials	**No change in activity** of cervical muscles relative to forward-directed pull trials
**Added head mass and rearward-directed force**	Increased forces cervical muscles must produce to maintain postural stabilization in forward-directed force trials	**Increase in activity** of cervical muscles relative to rearward-directed pull trials	**No change in activity** of cervical muscles relative to rearward-directed pull trials

A third result that does not support the postural stabilization hypothesis is the observation that when mass was added to the head, doubling its inertia, in combination with the added horizontal forces, activity of the cervical muscles did not increase (Figs. 2 and 3, Table 6). The one exception was the splenius capitis muscle which exhibited a 39% increase in activity when mass was added to the head in the forward-directed added force trials. However, for this muscle's increased activity to be associated with postural stabilization of the head the increase would have to occur in the rearward-directed added force trials. Although a larger subject sample might have revealed significant increases in muscle activity in those muscles in this experiment, these results are consistent with the possibility that the superficial cervical muscles monitored in this study do not play a large role in postural stabilization of the head during running.

### Cervical muscle activity during maximum effort countermovement jumps

**Fig. 4:**
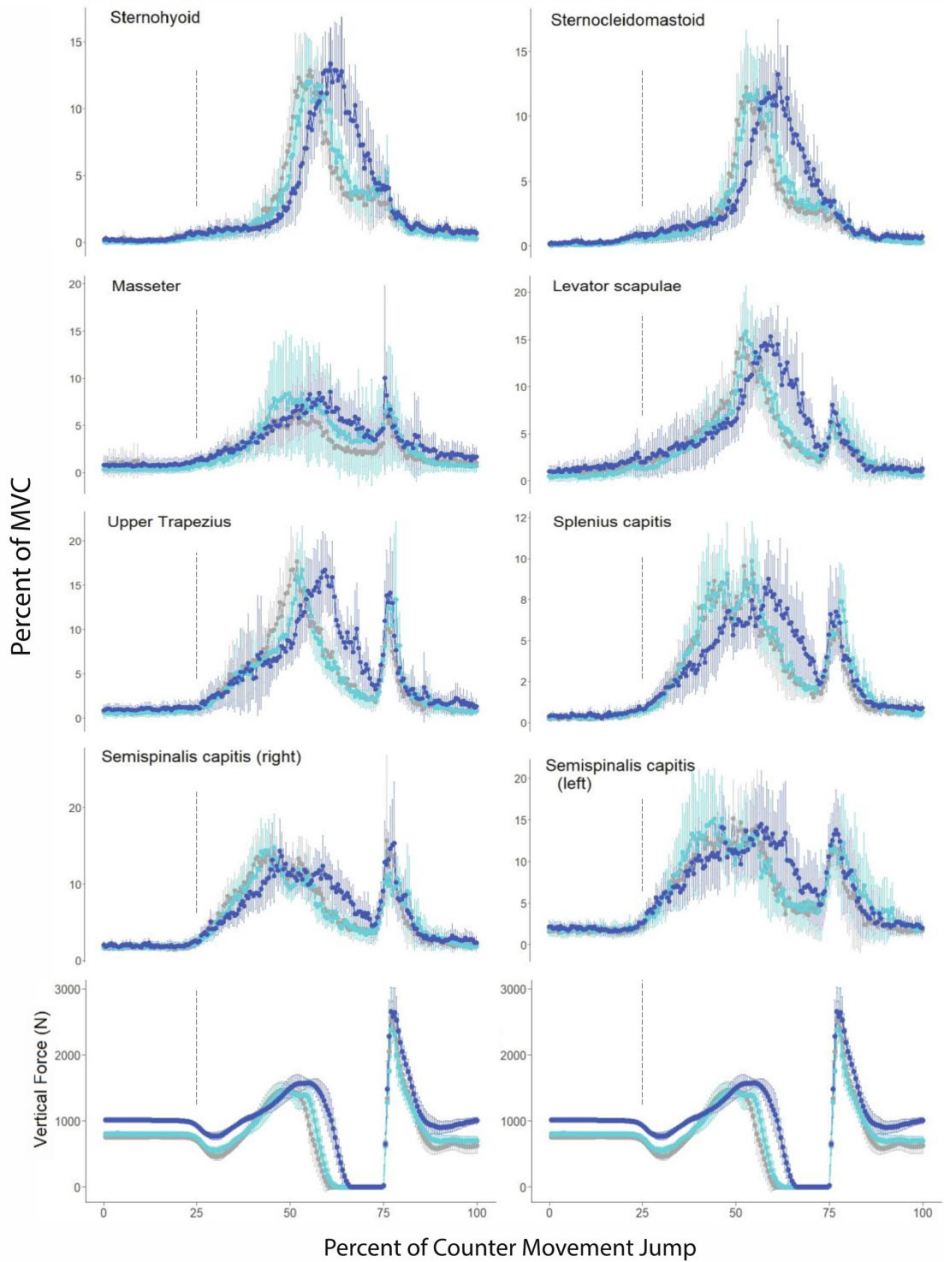
Plots of average muscle activity of subjects during countermovement maximum effort jumps, comparing the unencumbered control jumps (gray line), the reduced acceleration jumps (dark blue line), and the added head mass (light blue line). All muscles are on the right side of the neck unless noted otherwise. In each panel, the dashed vertical lines represent the start of the countermovement and touch-down from the jump, respectively. Values for the muscle activity are expressed as a mean % of MVC. Error bars represent 95% confidence intervals.

Activity of the epaxial muscles monitored in this study (splenius capitis and the semispinalis capitis), as well as the levator scapulae and upper trapezius muscles, which also exert an extensor moment on the neck, began after subjects initiated the drop phase of the countermovement jump. Activity continued throughout the power phase of the jump, reaching peak EMG amplitude approximately 100 ms before the peak ground force for the epaxial muscles and shortly after peak ground force for the levator scapulae and upper trapezius muscles (Fig. 4, Sup. Fig. 4). These muscles exhibited lower levels of activity during the flight phase of the jump and their activity increased again as landing from the jump became imminent. In contrast, activity of the sternohyoid, sternocleidomastoid, and masseter muscles began coincident with peak ground force and continued throughout the flight phase of the jump, activity then declined as the subjects landed. The five muscles that were active during the push-off phase of the jump increased their activity again shortly before touch down.

To test the hypothesis that cervical muscles contribute to the control of posture of the head during jumping we completed two force manipulations. First, to reduce the forces that the cervical muscles must produce to provide postural stabilization of the head, we increased the force of gravity by applying a downward directed force of approximately 270 Newtons via elastic bands attached to a waist harness. This manipulation reduced the acceleration force impulse applied by the subjects to the force plate by an average of 22%. Second, to increase the forces that cervical muscles must produce to provide postural stabilization to the head, we added 4.5 kg to the head. This added mass roughly doubled the inertia of a subject's head and reduced the force impulse applied to the force plate by only 3%.

Our manipulations of acceleration of the body and inertia of the head during maximum effort jumps did not change the activity of the cervical muscles (Fig. 4, Table 7). Notably, regardless of the metric compared, maximum voltage, mean voltage, or integrated area of the rectified EMG, the force manipulations did not change the level of muscle activity relative to the control trials. Activity of cervical muscles that control the posture of the head during acceleration of the body was predicted to decrease in maximum effort jumps in which acceleration of the body was reduced by increasing the downward directed force (Table 3). This was not observed. In contrast, activity of cervical muscles that stabilize the head during acceleration was predicted to increase when the mass of the head was substantially increased. Except for the trapezius muscle, activity of the cervical muscles did not change when the mass of the head was roughly doubled. Activity of the trapezius muscle actually decreased, rather than increased, when subjects jumped with mass added to the head. Because all jumps in the control trials and the force manipulation trials were maximum effort jumps, the core stabilization hypothesis leads to the prediction that cervical muscle activity would not differ between control and manipulation trials (Table 3). The experimental results are consistent with this prediction.

**Table 3:** Predictions for jumping experiments

Experiment	Force manipulation	Prediction of postural stabilization hypothesis	Prediction of core stabilization hypothesis
**Added head mass**	Increased forces cervical muscles must produce to maintain postural stabilization	**Increase in activity** of cervical muscles relative to control trials	**No change in activity** of cervical muscles relative to control trials
**Added downward-directed force**	Decreased forces cervical muscles must produce to maintain postural stabilization	**Decrease in activity** of cervical muscles relative to control trials	**No change in activity** of cervical muscles relative to control trials

### Cervical muscle activity during countermovement jumps of variable intensity

To document the extent to which cervical muscle activity is correlated with jumping effort we asked subjects to complete a series of 20 additional jumps over a range of efforts from 10 to 90% maximum. In these trials, integrated EMG was positively correlated with the jump force impulse (Supplementary Fig. 5). For each muscle, the best least-squared fit to the data was an exponential function, indicating that intensity of muscle activity tended to increase rapidly as jump effort increased.

## Discussion

### Interpretation of results from the running experiments

In the cervical muscles examined in this study, maximum and average activity during running unencumbered was of a relatively low level compared to maximum voluntary contraction, 0.4–6.0% and did not exceed 10% of MVC during the force manipulation trials. This indicates that the required forces from these muscles are relatively small during running. From the perspective of core stabilization, this result is not surprising given that the extrinsic muscles of the arm, some of which originate on cervical vertebrae and the skull, can be anticipated to impose much larger destabilizing moments on the cervical axial skeleton during routine human behaviors and therefore require much greater activation of cervical muscles for core stabilization.

To test the hypothesis that the cervical muscles we monitored assist in postural stabilization of the head during a running stride (Fig. 1A) we increased the mass of the head and compared muscle activity in the added mass trials to that of control trials. By roughly doubling the inertia of the head, without changing accelerations of body (recorded at the neck, Table 4), maintenance of head posture can reasonably be assumed to require an associated increase in force from the cervical muscles, perhaps a near doubling of muscle force. Thus, an observation of an increase in muscle activity with this manipulation would support the postural stabilization hypothesis (Table 2). Yet, activity of six of the eight muscles monitored in this study did not exhibit a significant increase relative to control trials when mass was added to the head. Only the levator scapulae and upper trapezius muscles exhibited relatively small increases, 21 and 26%, respectively, in the added head mass trials. Although a larger subject sample might have revealed significant increases in muscle activity, the results of this experiment are consistent with the possibility that the superficial cervical muscles monitored in this study do not play a large role in postural stabilization of the head during running.

**Table 4:** Mean vertical and fore-aft acceleration of the neck during steady-state running on a treadmill without (control) and with applied force manipulations

Force manipulation	Mean vertical acceleration (m ^.^ s^−2^)	Mean fore-aft acceleration (m ^.^ s^−2^)
**Control**	9.31 ± 0.96	2.27 ± 0.98^a^
**Added head mass**	9.11 ± 1.04 p = 0.30^*^	2.05 ± 1.16^a^*P* = 0.07^*^
**Forward pull**	–10.10 ± 1.66 p = 0.16^*^	–6.09 ± 1.55^b^*P* = 0.004^*^
**Forward pull plus added head mass**	–9.58 ± 1.45 p = 0.10^**^	–5.75 ± 1.98^b^*P* = 0.73^**^
**Rearward pull**	9.58 ± 1.79 p = 0.73^*^	6.65 ± 2.36^b^*P* = 0.004^*^
**Rearward pull plus added head mass**	8.88 ± 1.56 p = 0.004^***^	5.67 ± 1.92^b^*P* = 0.07^***^
**Comparison of mean acceleration during forward and rearward trials**	p = 0.30	*P* = 0.50

* *P*-value calculated relative to control trials.

** *P*-value calculated relative to forward pull trials.

*** *P* -value calculated relative to rearward pull trials.

a Average during accelerating portion (second half) of the step.

b Average during the entire step.

**Table 5:** Ratio of the integrated muscle activity recorded during the added head mass trials over that recorded during the control trials when subjects ran at 2.7 ms^−1^ on a treadmill

	N	Mean ± S.D.	*P*-value
**Sternohyoid**	16	1.24 ± 0.59	0.7
**Sternocleidomastoid**	16	1.30 ± 0.68	0.86
**Masseter**	12	0.71 ± 0.38	0.084
**Levator scapulae**	14	1.21 ± 0.27	0.18
**Upper Trapezius**	16	1.26 ± 0.24	**<0.008**
**Splenius capitis**	15	1.10 ± 0.26	0.86
**Semi spinalis (right)**	16	1.07 ± 0.15	0.7
**Semi spinalis (left)**	11	1.10 ± 0.27	0.7

Values are mean ± standard deviation, of the integrated area per locomotor cycle expressed as a ratio of activity during the control run.

*P*-values were calculated relative to control trials using the Holm-Bonferroni Sequential Correction.

In contrast to our finding that added head mass had little or no effect on cervical muscle activity during running, a study that monitored EMG of three cervical muscles (sternocleidomastoid, erector spinae, and trapezius) observed 18 to 28% increases in peak muscle activity in response to wearing an aviation helmet during trampoline exercises that induced accelerations up to 4 G (Sovelius et al., 2008). We suspect this difference is due to the larger accelerations the subjects were exposed to by jumping on a trampoline. (Note, peak acceleration experienced by our subjects during the countermovement jump trials did not exceed 2 G, discussed below.)

To test the core stabilization hypothesis (Fig. 1B), we increased the flexion and extension moments at the hip joint by applying horizontal forward and rearward directed forces with elastic bands. By increasing fore and aft accelerations of the body, application of horizontal forces to running subjects has dual effects of (1) increased fore-aft pitching moments imposed on the head, creating an increased need for postural stabilization and (2) increased flexion and extension torques applied at the hip joint by the extrinsic limb muscles, creating an increased need for core stabilization. Fortunately, which of these two effects influenced activity of the monitored muscles can be discerned because muscles providing postural stabilization to the head are located on the opposite side of the neck from muscles acting to stabilize the pelvis during a running stride (compare panels A and B in Fig. 1). Thus, if the sternohyoid, sternocleidomastoid, and masseter muscles are part of a linked chain of axial muscle activation that contribute to the musculoskeletal core of the pelvic girdle we predicted the greatest increases in their activity would occur during the forward pull trials to assist in stabilizing the pelvis against increased leg flexion torques. Similarly, if the cervical epaxial (semispinalis capitis and splenius capitis), the levator scapulae, and upper trapezius muscles contribute to core stabilization, we predicted they would exhibit their largest increase in activity during the rearward pull trials to assist in stabilization of the pelvis against extension torques (Table 2). Apart from the masseter muscle, our results are consistent with these predictions. The ventral cervical muscles displayed significantly greater activity during the forward pull trials than the rearward pull trials (Table 6). In a similar manner, dorsal muscle activity was greater in the rearward pull trials when compared to the forward pull trials. Thus, the superficial cervical muscles appear to assist in core stabilization against flexion and extension torques applied at the hip joint by the extrinsic muscles of the lower limb during running.

**Table 6:** Ratio of the integrated muscle activity recorded during the added horizontal force manipulated trials over that recorded during the control trials when subjects ran at 2.7 ms^−1^ on a treadmill

	Forward pull	Forward pull plus head mass	Rearward pull	Rearward pull plus head mass	Forward versus rearward trials
**Sternohyoid**	3.63 ± 2.24 (N = 16) ***P* < 0.001**	3.74 ± 1.98 (N = 10) *P* = 1	2.27 ± 1.57 (N = 16) ***P* < 0.001**	2.32 ± 2.21 (N = 10) *P* = 1	F > R ***P* = 0.01**
**Sternocleidomastoid**	2.74 ± 1.28 (N = 16) ***P* < 0.001**	2.65 ± 1.29 (N = 10) *P* = 1	1.72 ± 0.68 (N = 16) ***P* < 0.02**	1.59 ± 0.84 (N = 10) *P* = 1	F > R ***P* = 0.01**
**Masseter**	1.53 ± 1.12 (N = 12) *P* = 0.42	0.84 ± 0.61 (N = 6) *P* = 1	1.17 ± 0.52 (N = 12) *P* = 0.57	0.77 ± 0.52 (N = 6) *P* = 1	No difference *P* = 0.64
**Levator scapulae**	1.78 ± 0.51 (N = 14) *P* < 0.05	1.96 ± 0.80 (N = 8) *P* = 1	2.15 ± 0.70 (N = 14) ***P* < 0.004**	2.18 ± 0.76 (N = 8) *P* = 1	No difference *P* = 0.24
**Upper Trapezius**	1.23 ± 0.37 (N = 16) *P* = 0.12	2.03 ± 2.24 (N = 10) *P* = 0.42	1.96 ± 0.60 (N = 16) ***P* < 0.001**	1.91 ± 0.72 (N = 10) *P* = 1	F < R ***P* < 0.008**
**Splenius capitis**	1.50 ± 0.38 (N = 15) ***P* < 0.006**	2.08 ± 1.26 (N = 9) *P* = 0.08	2.01 ± 0.65 (N = 15) ***P* < 0.001**	2.13 ± 0.82 (N = 9) *P* = 1	F < R ***P* = 0.008**
**Semi spinalis (right)**	1.33 ± 0.32 (N = 16) *P* < 0.05	1.37 ± 0.34 (N = 10) *P* = 1	1.82 ± 0.45 (N = 16) ***P* < 0.001**	1.84 ± 0.52 (N = 10) *P* = 1	F < R ***P* < 0.008**
**Semi spinalis (left)**	1.29 ± 0.25 (N = 11) *P* < 0.05	1.68 ± 0.44 (N = 5) *P* = 0.42	1.80 ± 0.41 (N = 11) ***P* < 0.004**	1.87 ± 0.28 (N = 5) *P* = 1	F < R ***P* = 0.01**

Values are mean ± standard deviation, of the integrated area per locomotor cycle expressed as a ratio of activity during the control run.

*P*-values were calculated using the Holm–Bonferroni Sequential Correction relative to control trials for the forward pull and rearward pull trials; relative to the forward pull trials for the forward pull plus head mass trials; and relative to rearward pull for the rearward pull plus head mass trials.

**Table 7:** Maximum voltage, mean voltage, and integrated area of the rectified EMG of the manipulated trials presented as a proportion of the control trials when subjects executed maximum effort countermovement jumps

		Added Gravity			Added head mass		
	N	Maximum voltage	Mean voltage	Integrated area	Maximum voltage	Mean voltage	Integrated area
**Sternohyoid**	16	1.00 ± 0.21 (p = 1)	1.23 ± 0.29 (p = 0.324)	0.92 ± 0.20 (p = 1)	1.00 ± 0.21 (p = 0.695)	1.00 ± 0.21 (p = 1)	1.00 ± 0.21 (p = 1)
**Sternocleidomastoid**	16	0.97 ± 0.15 (p = 1)	1.18 ± 0.31 (p = 0.308)	1.06 ± 0.27 (p = 0.978)	0.94 ± 0.22 (p = 0.623)	1.03 ± 0.29 (p = 1)	1.06 ± 0.27 (p = 1)
**Masseter**	11	1.20 ± 0.52 (p = 1)	1.25 ± 0.47 (p = 0.748)	1.15 ± 0.48 (p = 1)	0.83 ± 0.38 (p = 0.623)	0.85 ± 0.39 (p = 0.637)	0.86 ± 0.39 (p = 0.39)
**Levator scapulae**	12	1.08 ± 0.28 (p = 1)	1.22 ± 0.46 (p = 0.28)	1.10 ± 0.41 (p = 1)	1.04 ± 0.32 (p = 0.864)	1.10 ± 0.51 (p = 1)	1.13 ± 0.53 (p = 1)
**Upper trapezius**	16	1.19 ± 0.42 (p = 0.784)	1.09 ± 0.29 (p = 0.748)	0.99 ± 0.25 (p = 1)	0.94 ± 0.39 (p = 0.136)	0.92 ± 0.17 (p = 0.056)	0.95 ± 0.14 (p = 0.136)
**Splenius capitis**	16	0.94 ± 0.28 (p = 0.819)	1.03 ± 0.23 (p = 0.748)	0.93 ± 0.20 (p = 0.889)	1.10 ± 0.40 (p = 0.864)	1.06 ± 0.22 (p = 1)	1.10 ± 0.18 (p = 0.343)
**Semi spinalis (right)**	16	0.98 ± 0.24 (p = 1)	1.03 ± 0.17 (p = 0.748)	0.94 ± 0.18 (p = 0.536)	0.97 ± 0.32 (p = 0.695)	0.94 ± 0.17 (p = 0.637)	0.98 ± 0.17 (p = 1)
**Semi spinalis (left)**	10	1.03 ± 0.31 (p = 1)	1.11 ± 0.23 (p = 0.345)	1.01 ± 0.20 (p = 1)	0.92 ± 0.28 (p = 0.695)	0.99 ± 0.20 (p = 1)	1.00 ± 0.18 (p = 1)

Values are mean ± standard deviation, expressed as a ratio of activity during the control jumps.

*P*-values as shown in parentheses and were calculated using the Holm–Bonferroni Sequential Correction relative to control trials.

Given established patterns of the moments that occur at the hip joint during running, specific predictions can be made regarding the phase relationships of increased muscle activity that would support the core stabilization hypothesis. In our trials, acceleration occurred during the entire stance phase when subjects were pulled rearward (Table 4). When accelerating, the GRF vector is directed forward creating the greatest ground force moment arm at the hip joint during the beginning of the step which is accompanied by larger hip joint moments compared to the end of the stance phase (Roberts and Bellveau, 2005; Bezodis et al., 2014). Consistent with this, the greatest increase in activity of the cervical epaxial muscles coincided with the first half of the step in our rearward pull trials. Conversely, when subjects were pulled forward they were forced to decelerate during the entire step (Table 6). During deceleration, the ground reaction force is directed rearward and the orthogonal distance between the hip joint and GRF vector, is greatest during the second half of the step and smallest during the first half of stance. This results in larger hip joint moments during the second half of braking steps (Kuster et al., 1995). From this, one would predict that the greatest increase in activity of the cervical hypaxial muscles would coincide with the second half of the step during forward pull trials, which is also what we observed.

One surprising result of this study is the observation that activity of the cervical muscles we monitored did not exhibit significant increases in activity when mass of the head was doubled during the added forward- and rearward-directed force trials (Figs. 2 and 3, Table 6). This result is consistent with the possibility the superficial cervical muscles monitored in this study do not play an important role in postural stabilization of the head during running and mirrors the results obtained when the mass of the head was doubled during normal running and maximum effort counter movement jumps. However, in this case, the limited sample sizes of 10 or fewer subjects increases the possibility that variance in the data swamped the ability of the statistic to detect a difference.

The result we did not anticipate was the increase in activity in ventral strap muscles during rearward pull trials and in the dorsal muscles of the neck during forward pull trials. These increases were less than those observed in the trials in which we expected to see increased activity, but nonetheless were significant increases relative to control trials. We suspect that these tonic increases in muscle activity represent coactivation of antagonistic muscles that are fundamental to core stabilization. For example, if epaxial muscles of the neck are part of the linked-chain of dorsal muscle activation that stabilizes the pelvis during limb extension (i.e., acceleration of the body), coactivation of the ventral strap muscles of the neck is likely required to avoid neck extension. The observed lower coactivation of muscles on the opposite side of the neck may be necessary to “ground” the head and neck to the torso to eliminate head movements that would interfere with sensory perception.

### Interpretation of results from the jumping experiments

During vertical jumping, the cervical muscle moments needed for postural stabilization of the head temporally coincide with those needed for core stabilization (Fig. 1 A, B). For this reason, to distinguish between these two functional roles for a given muscle we manipulated the cervical moments needed for postural stabilization of the head by (1) reducing acceleration of the body and (2) doubling the mass of the head while holding the hypothesized cervical moments necessary for core stabilization constant by asking subjects to exert maximum effort in each of their countermovement jumps. The force needed from the neck muscles to control head posture during acceleration of the body is the product of the mass of the head multiplied by the acceleration of the head. By reducing the acceleration of the body with increased downward directed force, the force needed from the cervical muscles for postural stabilization of the head would be reduced and we would expect a corresponding reduction in EMG activity. In contrast, by doubling the mass of the head, the force needed from the cervical muscles for postural stabilization of the head during the jump should increase substantially, and we would expect to see a large increase in muscle activity (Table 3). Our results suggest that neither of these expectations for postural stabilization of the head are valid for the muscles investigated in this study. Thus, both our running and jumping results do not support the hypothesis that the superficial muscles of the neck play an important role in the postural stabilization of the head during locomotion.

Because subjects jumped with maximal effort during both the control and manipulation (added gravity, and added head mass) trials, the torque applied at the hip joint by the extrinsic muscles of the leg should be roughly the same in each trial. If the torque at the hip joint is the same across all manipulations, the muscle moments required for core stabilization are expected to also be roughly the same in all jumps. Therefore, the core stabilization hypothesis predicts that activity of the cervical muscles needed to resist the torque applied at the hip joint should also not change in the force manipulation trials compared to the control trials (Table 3). This is what we observed. Thus, both our running and jumping results are consistent with the hypothesis that the superficial muscles of the neck function as part of the linked-chain of muscle activation responsible for core stabilization of the pelvis during locomotion.

### Why do the cervical muscles investigated in this study appear not to be involved in postural stabilization of the neck and head?

In both the running and jumping force manipulation experiments we found little evidence of changes in muscle activity that are consistent with the investigated muscles contributing to postural stabilization of head. This is surprising given that there are eight intervertebral joints between the skull and the first thoracic vertebrae and the manipulation of doubling the mass of the head should require roughly a doubling of force from the muscles responsible for postural stabilization. Clearly, cervical muscles must be activated to provide postural stabilization of the head during running and jumping, however, our results suggest that the superficial cervical muscles that we monitored with surface EMG are not those muscles.

Motor control may require a division of labor in the cervical muscles because postural stabilization of the head sometimes requires muscle activation on the opposite side of the neck from muscle activation that is simultaneously required for core stabilization (Fig. 1 A and B). Based on topology, axial muscles (lumbar, thoracic, and cervical) have been categorized into three broad functional categories: local stabilizers, global stabilizers, and global mobilizers (Panjabi et al., 1989; Hides et al., 1996; Schilling, 2009; 2011). The local stabilizers are the deep, mono-segmental muscles, that are suggested to work largely eccentrically and exhibit continuous activity in order to maintain and control segmental stability. Global stabilizers are suggested to control the range of movement of the spine and exhibit motion-dependent activity. The third group, global mobilizers are suggested to contract concentrically and be largely responsible for the positive work of the trunk. Although this hypothesis has not been fully tested, studies of the distribution of muscle fiber types in the paravertebral muscles of small mammals (Schilling, 2009) and other vertebrates (Schilling, 2011) have demonstrated greater concentration of slow-oxidative fibers in deeper, more medially located muscles and a higher percentage of fast glycolytic fibers in the more superficial lumbar epaxial muscles; a pattern consistent with the suggestion that the deeper muscles play a larger role in the control of postural stabilization than do the more superficial muscles. Interestingly, this pattern was not observed by the same research group in an analysis of human lumbar paravertebral muscles (Hesse et al., 2013). In any case, the suggestion that functional divisions of labor exist in the axial muscles is not novel and may support our results that superficial cervical muscles are not playing a primary role in maintaining head posture during running and jumping.

We anticipate that the maximum cervical muscle moments needed for postural stabilization of the head are generally substantially lower than the cervical muscle moments routinely needed for core stabilization during forceful action by the arms or legs. If this is true, and postural stabilization of the head at times requires activation of different cervical muscles than those needed for core stabilization (e.g., Fig. 1 A and B), then mechanically it follows that core stabilization may be primarily accomplished by the superficial cervical muscles that have larger moment arms. Whereas postural stabilization of the head may be largely the responsibility of the deeper cervical muscles such as the longus colli and capitis, rectus capitis lateralis and posterior, intertransversarii colli anterior and posterior, semispinalis cervicis and thoracis, splenius cervicis, multifidus, iliocostalis cervicis, cervical interspinal, longissimus cervicis and capitis, and obliquus capitis superior and interior.

Finally, motor control may also require a division of labor in the cervical muscles associated with postural versus core stabilization. The sensory modalities that control head posture are primarily vision, vestibular, and cervical proprioception (Di Fabio & Emasithi, 1997; Chiba et al., 2016). Motor control of core stabilization relies on proprioceptive feedback from a variety of mechano-receptors including muscle spindles, Golgi tendon organs, and joint receptors (McGill et al., 2003; Borghuis et al., 2008) that are located throughout the axial musculoskeletal system. Given these different sensory modalities, effective motor control may be facilitated by a division of labor in the cervical muscles such that some are primarily associated with postural control of the head whereas others are primarily responsible for cervical core stabilization.

### Limitations of the study

A limitation of our analysis is that the equipment available for this investigation did not allow simultaneous recording of ground reaction forces during the running trials. Consequently, although our intent with the added horizontal pulling forces during running trials was to manipulate the muscle moments required at the hip joint, we could not document the extent to which hip joint moments changed due to the force manipulations. Nevertheless, the literature provides quantification of hip joint moments in analogous conditions to our horizontal force manipulations. A study that compared incline running to running on a horizontal treadmill with applied forward or rearward pulling forces found that the pulling forces are effective in simulating incline running for kinematic and kinetic variables, leg muscle activity, and oxygen consumption (Gimenez et al., 2014). Although these authors did not quantify muscle moments, the variables they did measure strongly support our assertion that incline running impacts joint moments in a manner similar to the horizontal force manipulations employed in this investigation. Thus, the finding that extensor moments at the hip joint increase dramatically during walking and running uphill (Roberts & Belliveau, 2005; Nuckols et al., 2020) suggests that the added rearward pulling force manipulation used in this study also increased the hip extensor moment. Similarly, forward acceleration during sprinting, also analogous to our rearward pull manipulation, is associated with significant increases in the extensor moments at the hip joint (Bezodis et al., 2014). In contrast, walking downhill, which is analogous to our forward pull manipulation, substantially increases the hip flexor moment, and the negative work and power produced by hip flexor muscles (Kuster et al., 1995; Park et al., 2019). Consistent with these observed changes in hip joint moments, several studies have documented increased activity in muscles that produce extensor moments at the hip joint when subjects walk and run uphill ( Wall‐Scheffler et al., 2010; Franz & Kram, 2012; Vernillo et al., 2017) and accelerate during sprinting (Bartlett et al., 2014). Finally, two hip extensor muscles, gluteus maximus and biceps femoris, have been shown to increase activity when subjects walked and ran with an added rearward directed force and activity decreased when the subjects walked and ran with an added forward directed force (Ellis et al., 2014). These observations, combined with the changes in acceleration recorded at the neck in our subjects (Table 4), give us confidence that our horizontal force manipulations increased the moments at the hip joint in the intended direction: increased extensor moments in the rearward pulling trials and increased flexor moments in the forward pulling trials.

A second limitation of the study is that the setting of the high-pass filters of the AC amplifiers at 100 Hz eliminated a significant portion of surface EMG signal. Surface EMG signals range from 0.5 to 400 Hz (Komi & Tesch, 1979; Basmajian & De Luca, 1985; De Luca et al., 2010). Recommended settings of high-pass filters of surface EMG range from 10 to 20 Hz (Merletti & Di Torino, 1999; De Luca et al., 2010). The available literature suggests that several of the cervical muscles we recorded from have median surface EMG frequencies close to 100 Hz (Kumar et al., 2001). This means that with our filter setting of 100 Hz we effectively filtered away half of the signal. Although this mistake is problematic, there are several arguments that give us confidence that the range of frequencies we recorded does provide a valid assessment of the muscles’ responses to the force perturbations.

Our understanding is that the range frequencies in EMG is determined by (1) variation in the conduction velocity of the muscle fibers, (2) distance between the electrodes (this is large for surface electrodes, producing the low range of recorded frequencies), and (3) the adding and cancelling of frequency components due to fact that signal is a compound action potential. A large proportion of the lower and upper frequency elements in EMG are likely a result of frequency addition and cancelling. This is important because, if true, it suggests that the lowest frequencies we filtered out of the signal are unlikely to provide unique information about the muscles’ responses to the force manipulations.

Our main concern with our data is the possibility that the response to our force manipulations of the slow oxidative fibers, which produce lower frequencies, may have been different than that of the fast glycolytic fibers, which produce higher frequencies. If the low frequencies we removed are more representative of signals produced by slow fibers this would bias the interpretation of the results. However, in most of the experiments, the manipulations were intended to increase the forces that the cervical muscles needed to produce. As the required force a muscle must produce increases, recruitment generally proceeds from slow to fast fibers. Thus, to the extent we removed the signal of the slower fibers, the amplitude of the control trials should be diminished relative to the amplitude of the manipulation trials. In other words, our filter setting would be expected to increase the difference between the control trials and the manipulation trials rather than reduce the difference. Yet, in our added head mass manipulations, in which we roughly doubled the inertia of the head, we found little or no increase in EMG compared to the control trials. This was true in both the running and jumping experiments. Thus, our poor choice in filter setting, biased our recordings in the direction of seeing a difference between the control and added head mass trials, but we did not observe a difference.

It also warrants mention that the high-pass filters of our Grass amplifiers remove approximately 50% of the signal at the frequency selected, 100 Hz in this case, and approximately 75% at half that frequency, 50 Hz in this case. Thus, our recordings do include some portion of the lower frequencies produced by the muscles, down to at least 50 Hz. For these three reasons, we have confidence that our high cut-off frequency on the high-pass filters did not bias the data in a way that led to incorrect interpretations.

Finally, although doubling the mass of the head produced little or no evidence of increased muscle activity in both the running and jumping trials, the applied forward and reward directed horizontal forces resulted in dramatic increases in activity of the cervical muscles. This difference in muscle response to the two force manipulations gives us confidence that our recordings do provide a valid indication of the changes in muscle activity in response to the force manipulations.

A final limitation is that the sample size for the horizontal pull plus added head mass trials was lower (5–10) than that for the other trials (11–16). With one exception (Splenius capitis in the forward pull plus added head mass) these trails were found to not differ significantly from the horizontal pull trials. It is possible that with a larger sample size significant differences would have been observed.

### Broader implications

Our interest in the role of the human neck in locomotion was motivated, in part, by the question of whether activity of cervical muscles can provide protection against concussion injury due to blind side impacts such as those that often occur in contact sports (e.g., Ice hockey and American football). If muscles of the neck are routinely active during vigorous locomotion, they may provide intrinsic protection against concussion inducing impacts. By stiffening joints and absorbing energy, active muscle plays an important role in preventing injury of the limbs and spine (Baratta et al., 1988; Loeb, 1995; Burr, 2011). Most importantly for athletes in contact sports, co-activation of muscles of the neck and trunk may help prevent concussion by reducing acceleration of the head by linking the mass of the head to the mass of the whole body (Viano et al., 2007; Hasegawa et al., 2013; Eckner et al., 2014). However, for muscles to provide this protection they need to be activated and generating force prior to impact because reflexes are much too slow to provide protection during impact. In collegiate football, average duration of head impacts is 14 ms, with peak linear accelerations occurring 7 to 11 ms after contact (Rowson et al., 2009)^.^ However, the stretch reflex latency of cervical muscle electrical activation ranges from 51 to 60 ms (Corna et al., 1996) and the temporal delay between electrical activation and generation of measurable force (force-activation delay) is approximately 50 ms in human muscles (Cavanagh & Komi, 1979). This means that reflexes are roughly ten-times slower than needed for protection. Thus, cervical muscles need to be actively generating force prior to impact to be protective which is unlikely to occur in instances when a player is blindsided, unless cervical muscle activity is intrinsic to locomotion. Our results suggest that locomotor activity of the cervical muscles for core stabilization may offer a level of protection to the cervical spine and possibly the brain against unanticipated impacts. All else being equal, the results of this study suggest that the risk of injury from unanticipated impacts will be greatest when a person is stationary and that the level of intrinsic protection increases as cervical muscle activity increases with the intensity of the locomotor performance.

## Supplementary Material

obac021_Supplemental_Videos
